# The Role of a Novel *TRMT1* Gene Mutation and Rare *GRM1* Gene Defect in Intellectual Disability in Two Azeri Families

**DOI:** 10.1371/journal.pone.0129631

**Published:** 2015-08-26

**Authors:** Behzad Davarniya, Hao Hu, Kimia Kahrizi, Luciana Musante, Zohreh Fattahi, Masoumeh Hosseini, Fariba Maqsoud, Reza Farajollahi, Thomas F. Wienker, H. Hilger Ropers, Hossein Najmabadi

**Affiliations:** 1 Genetics Research Center (GRC), University of Social Welfare and Rehabilitation Sciences, Tehran, Iran; 2 Department of Human Molecular Genetics, Max-Plank Institute for Molecular Genetics, Berlin, Germany; 3 Welfare Organization of Ardabil Province, Ardabil, Iran; Hadassah-Hebrew University Medical Center, ISRAEL

## Abstract

Cognitive impairment or intellectual disability (ID) is a widespread neurodevelopmental disorder characterized by low IQ (below 70). ID is genetically heterogeneous and is estimated to affect 1–3% of the world’s population. In affected children from consanguineous families, autosomal recessive inheritance is common, and identifying the underlying genetic cause is an important issue in clinical genetics. In the framework of a larger project, aimed at identifying candidate genes for autosomal recessive intellectual disorder (ARID), we recently carried out single nucleotide polymorphism-based genome-wide linkage analysis in several families from Ardabil province in Iran. The identification of homozygosity-by-descent loci in these families, in combination with whole exome sequencing, led us to identify possible causative homozygous changes in two families. In the first family, a missense variant was found in *GRM1* gene, while in the second family, a frameshift alteration was identified in *TRMT1*, both of which were found to co-segregate with the disease. GRM1, a known causal gene for autosomal recessive spinocerebellar ataxia (SCAR13, MIM#614831), encodes the metabotropic glutamate receptor1 (mGluR1). This gene plays an important role in synaptic plasticity and cerebellar development. Conversely, the *TRMT1* gene encodes a tRNA methyltransferase that dimethylates a single guanine residue at position 26 of most tRNAs using S-adenosyl methionine as the methyl group donor. We recently presented *TRMT1* as a candidate gene for ARID in a consanguineous Iranian family (Najmabadi et al., 2011). We believe that this second Iranian family with a biallelic loss-of-function mutation in *TRMT1* gene supports the idea that this gene likely has function in development of the disorder.

## Introduction

Cognitive impairment or intellectual disability (ID) is estimated to affect up to 3% of the general population and can be caused by both environmental and genetic factors, such as chromosomal aberrations or autosomal recessive, dominant, X-linked or mitochondrial mutations**.** ID can be divided into two main groups: non-syndromic (NS) ID, where it might present with ID without additional features, and syndromic ID, in which additional clinical or dysmorphic features may also be present [1, 2, 3].

Studies of the molecular basis of intellectual disability have focused on X-linked ID in part, because the larger families which is needed for gene mapping in ARID are rare in Western countries. A recent review suggests that ARID is not rare and in outbred populations, 13–24% of ID may be due to AR genes [4]. In populations where parental consanguinity is common, autosomal recessive gene defects are an even more important cause of ID. In families from the Middle East, autosomal recessive disorders were found to be approximately three times more frequent among inbred *vs*. non-inbred families [5].

Although mutations in approximately 1000 different genes may cause ID [6], it is thought that there are no molecular diagnoses for up to 50% of ID cases at present [7]. For the non-syndromic form of ID, it may be difficult to pinpoint and detect the molecular cause of some cases with minor clinical features unless a candidate gene is found in more than one individual.

In Iran, the rate of consanguineous marriages is about 40%, and the sharing of founder mutations between large related affected families could allow the identification of disease-associated genes. Due to this great resource, the Genetics Research Center (GRC) was able to discover more than eight ARID-associated loci (MRT4-11) between 2007 and 2011 [8,9]. Since 2011, using next-generation sequencing (NGS), we have identified 50 novel genes for ARID [10].

In this study, we present two novel variants in *GRM1* and *TRMT1* gene and propose that they are the underlying genetic causes of ARID in two Iranian families. Mutations in *GRM1* gene are responsible for spinocerebellar ataxia, autosomal recessive 13 (SCAR13; OMIM#614831; Guergultcheva et al., 2012). *GRM1* encodes a metabotropic glutamate receptor1 (mGluR1) that participates in long-term potentiation in the hippocampus and long-term depression in the cerebellum [11].

The second candidate gene we propose for ARID is *TRMT1*. In a recent study by Najmabadi et al., they recognize a family with *TRMT1* gene mutation with clinically similar symptoms and features to our family 9000114 in this study. While functional analyses of new candidate genes and associated mutations can provide support for the involvement of a candidate gene, perhaps the most convincing evidence for causality is to find matching cases, as we did here. *TRMT1* gene encodes a tRNA methyltransferase that dimethylates a single guanine residue at position 26 of most tRNAs [12]. Two RNA-methyltransferases, *FTSJ1* (MRX9, MIM#309549) and *NSUN2* (MRT5, MIM#611091), which have been identified previously, are implicated in X-linked ID and ARID, respectively, suggesting an important role of RNA methylation in cognition process [13, 14].

Overall, our results suggest that novel allelic variants of a large number of genes could lead to identification of Iranian patients affected with cognitive impairments. They also highlight how studying consanguineous families could help in discovering the genetic causes of heterogeneous disorders.

## Methods

We identified 25 consanguineous families with two or three affected individuals from Ardabil province, Iran. The study was approved by the ethics committee of the University of Social Welfare and Rehabilitation Sciences in Tehran, Iran.The parents, guardians, and individuals in this manuscript have given written informed consents (as outlined in PLOS consent form) to publish these case details and their pictures. All data created during this research are openly available from Genetics Research Center data archive. After obtaining written informed consent, the probands were examined by experienced clinical geneticists, who assessed the physical and mental status of the participants, and the participants then underwent a brain MRI to screen for abnormal anatomical features. The Wechsler Intelligence Scale for Children (WISC) was used to assess the IQ (intelligence quotient) of the patients. To rule out chromosomal aberrations and fragile X-syndrome, karyotype analysis by G-banding and fragile X testing by Southern blot analysis and PCR were performed. The karyotype of all patients was normal, and fragile X-syndrome was excluded. Genomic DNA was isolated from whole blood by following the standard salting out isolation method.

### SNP genotyping and linkage analysis

A whole genome scan was performed using SNP Array 6.0 (Affymetrix, Santa Clara, CA, USA) for two or three affected individuals, one unaffected individual and their parents, and the data were analysed using Homozygosity Mapper software[15,16]. We applied the ALOHOMORA [17] software v0.32 which use for converting genotyping data into appropriate format for linkage analysis. The gender-check tool of the ALOHOMORA software was also used to check the gender of selected individuals. Recognition of incompatibility genotypes from genotype data was performed using PedCheck software [18]. Graphical representation of relationship errors software or GRR [19] was used to calculate the relationships between individuals within families. The LOD score was calculated using the MERLIN program based on autosomal recessive mode of inheritance and with complete penetrance and a disease allele prevalence of 0.001 [20]. The threshold in this research for assessment of the intervals was a LOD score of approximately 2.5. The visualizations of haplotypes in linkage intervals were obtained using Haplopainter software.

### Whole Exome Sequencing and variant calling

The DNA of the index patients (IV.1 and V.2 for families 9000105 and 9000114, respectively) was used to generate an Illumina Pair End pre-capture library (SureSelect XT Target Enrichment System for Illumina Paired-End Sequencing Library; Agilent Technologies Inc., Wilmington, DE, USA) in accordance with the manufacturer's protocol (Agilent Technologies). The captured library was sequenced using the HiSeq 2000 (Illumina) in accordance with the manufacturer's protocol, in a 101-nucleotide single-end sequencing format. The selected exons were covered by an average depth of 100X, with 98% of them covered by at least 20X. To detect homozygous variants, we aligned the high-quality reads to the human reference genome GRCh37/hg19 by SOAP2.20. We also filtered out the neutral variants by matching dbSNP137, the 1000 Genome Project, ESP6500 exomes, and the 200 Danish exomes and were absent in Iranian controls [21,22]. Variants were ranked as potential candidates as previously described (Najmabadi et al., 2011) using an improved version of Medical Re-sequencing Analysis Pipeline (MERAP) developed at the Max Planck Institute for Molecular Genetics, Berlin, Germany. In addition, a pathogenic evaluation of the novel mutations was carried out according to present guidelines [23, 24].

The evaluation was based on several indicators: the effect of the mutation on the codon, *in silico* prediction of the functional effect at the amino acid level, the functional role of the gene related disease, brain-expression profile, evolutionary conservation, gene ontology (GO), and analysis of animal models, if available.

Candidate variants were validated in affected and normal individuals, and their parents, if available, by conventional Sanger sequencing.

## Results

Follow-up studies focused on 25 large consanguineous families from Ardabil province in Iran with two or three affected individuals. Here, we present two of these families in which autozygosity mapping and NGS led to the identification of a homozygous 2 bp deletion in *TRMT1* gene (OMIM*611669) and a homozygous missense change in *GRM1* gene (OMIM#604473).

**Fig 1:**
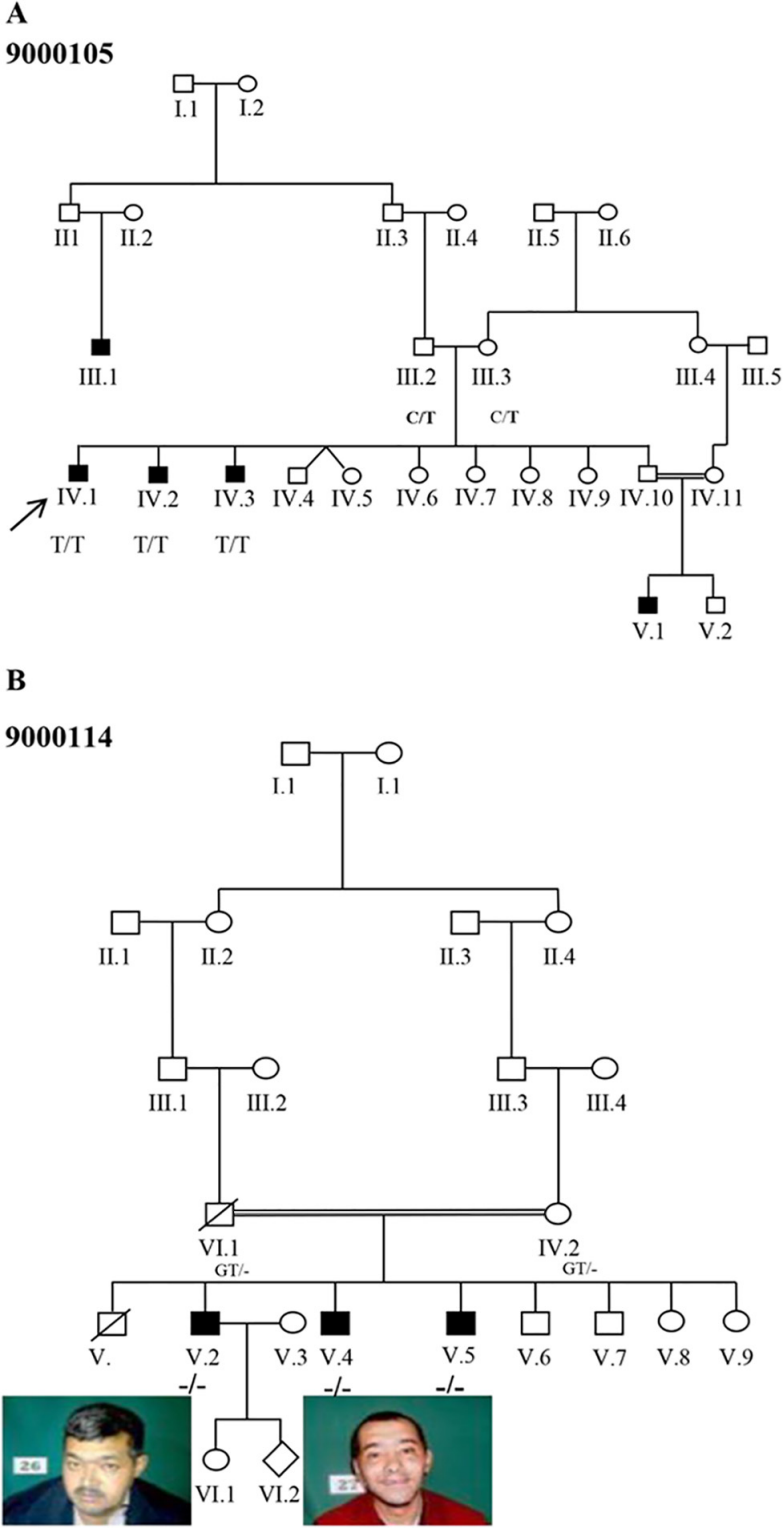
Pedigree of the Iranian family. (A) Pedigree of the family 9000105 with c.1360C>T; p.Leu454Phe mutation (B) Pedigree structure of the family 9000114 and facial appearance of the affected individuals with c.1332_1333delGT; p. Tyr445Leufs*28 mutation. Full symbols denote affected individuals with ID.

The pedigree of the families are shown in Fig 1. The clinical findings are summarized in Tables 1 and 2. The parents were clinically healthy; those deceased or unavailable were described as symptom free, supporting the autosomal recessive mode of inheritance.

**Table 1:** Clinical features of affected individuals from family 9000105 with a mutation in the *GRM1* gene.

Family	Family 9000105			Families (Guergueltcheva et al., 2012)
Individuals	IV:1	IV:2	IV:3	
GRM1 mutation (NM_001114329)	c.1360C>T, p. Leu454Phe	c.1360C>T, p.Leu454Phe	c.1360C>T, p.Leu454Phe	c.2652_2654del, (p.Asn885del) c.2660+2T>G‏
Gender	Male	Male	Male	Males and Females
Parental consanguinity	No	No	No	No
Origin	Iran, Ardabil province	Iran, Ardabil province	Iran, Ardabil province	Five Roma originating from a small subisolate group
Age at examination (yrs)	28	37	40	6 to 57
Age at simple sentences	10	11	11	2 to 4
Height (cm)	150(-4 SD)	150 (-4 SD)	150 (-4 SD)	120–154
Weight (Kg)	55(-2.5 SD)	58(-1.5 SD)	60(-1.2 SD)	22–60
Head circumference (cm)	55(+1 SD)	54(+0.5 SD)	54(+0.5 SD)	No data
Ataxia	Yes	Yes	Yes	Yes
Intellectual disability	Severe (IQ = 40)	Severe (IQ = 40)	Moderate (IQ = 55)	Mild-to-severe
Gait (0–8)	7	7	7	4 to 8
Stance	6	6	5	2 to 6
Seizure	At 1 year	At six month	At 1–2 years	Variable
Oculomotor Signs	Esotropia, Nystagmus	Nystagmus	Nystagmus	Nystagmus, Esotropia, Abduction deficit
Aggressive behaviour	Yes	Yes	Yes	No data

**Table 2:** Clinical features of affected individuals from family 9000114 with a new mutation in the *TRMT1* gene and from family M300 previously reported (Najmabadi et al., 2011).

Family	Family 9000114			Family M300	
Individuals	V:2	V:4	V:5	IV:4	IV:10
*TRMT1* mutation (NM_001136035)	c.1332_1333 delGT, p.Tr445Leu fs*28	c.1332_1333 delGT, p.Tr445Leu fs*28	c.1332_1333 delGT, p.Tr445Leu fs*28	c.657_688 del32, p.Gln219His fs*22	c.657_688 del32, p.Gln219His fs*22
Gender	Male	Male	Male	Male	Female
Parental consanguinity	First cousin	First cousin	First cousin	First cousin	First cousin
Origin	Iran, Ardabil province	Iran, Ardabil province	Iran, Ardabil province	Iran, Ahvaz province	Iran, Ahvaz province
Age at examination (yrs.)	49	33	26	24	11
Height (cm)	158 (>3% percentile)	159 (>3% percentile)	150 (-4 SD)	167 (+1 SD)	123
Head circumference (cm)	55 (+0.7 SD)	54 (+0.5 SD)	54 (+0.5 SD)	57 (+1 SD)	52 (-1 SD)
Weight	54 (-2 SD)	65 (0.0 SD)	51 (-2 SD)	No data	No data
Intellectual disability	Moderate (IQ = 55)	Mild (IQ = 70)	Moderate (IQ = 55)	Moderate	Moderate
Developmental delay	Yes	Yes	Yes	No	No
Facial dysmorphism	Synophrys broad nasal bridge, hypoplastic ma xilla	Synophrys, broad nasal bridge, hypoplastic maxilla	Synophrys, broad nasal bridge, hypoplastic maxilla	Protrudes ear, narrowing palpebral fissure and broad eyebrow	Protrudes ear, narrowing palpebral fissure and broad eyebrow
Involvements of the extremities	Yes	Yes	Yes	Yes	Yes
Others	-	Hearing loss; Polydipsia; Polyphagia	-	-	-

### Family 9000105

**Fig 2:**
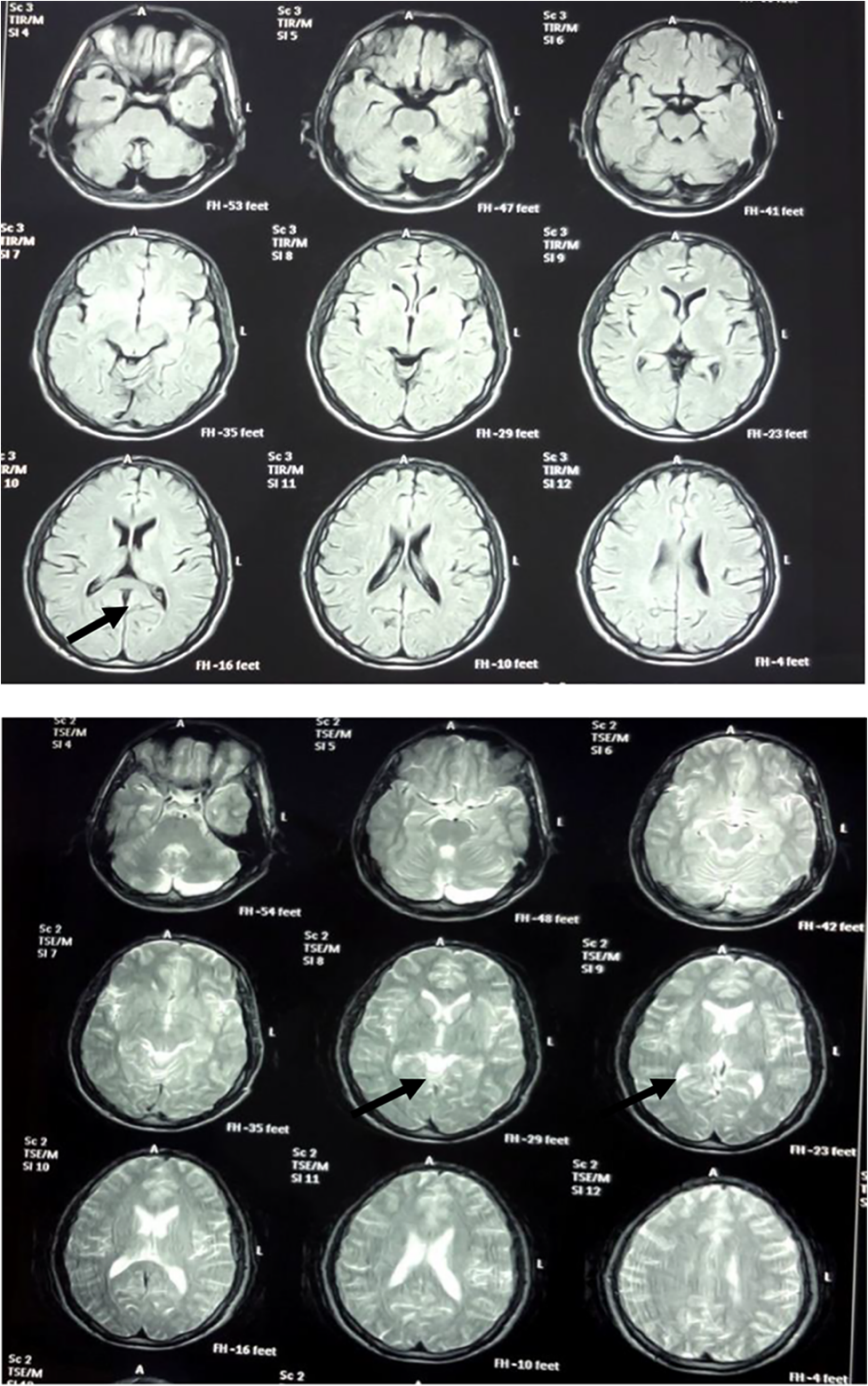
Braining imaging study. Brain imaging of the patient (IV: 4.1) with Congenital Cerebellar Ataxia; the images in T1 and T2 show cerebellar atrophy and severe increased number of cerebellar cisterns.

The investigated patients (IV.1, IV.2, and IV.3) were born at term with uneventful pregnancies. Their head circumferences and weights at birth and later during infancy were reported not to be in the normal range (50^th^ centile). All affected individuals had short stature (Table 1). The patients presented moderate-to-severe ID. All affected individuals had delayed developmental milestones and derangement of speech. An important neurologic symptom was ataxia, which were scaled in gait and stance. In a clinical examination to assay oculomotor signs, we found that patient IV.1 presented with nystagmus and esotropia while the other siblings were only affected by nystagmus. Frequent convulsions and aggressive behaviour were also observed. In patient IV.1, brain MRI revealed a severely increased number of cerebellar cisterns and cerebellar atrophy (Fig 2).

### Family 9000114

The patients in this family were born preterm and had low birth weight (LBW). They all showed developmental delay. Both gait and the ability to sit were reported to be delayed by 2 years in all of the affected. Although the speech ability was normal, it started between four and six years of age. The patients did not manifest any other neurological problems or congenital malformations except for mild facial dysmorphism. In addition, they showed weakness in the upper and lower extremities. Brain imaging did not yield any significant issues.

### Genotyping and Whole Exome Sequencing data

**Fig 3:**
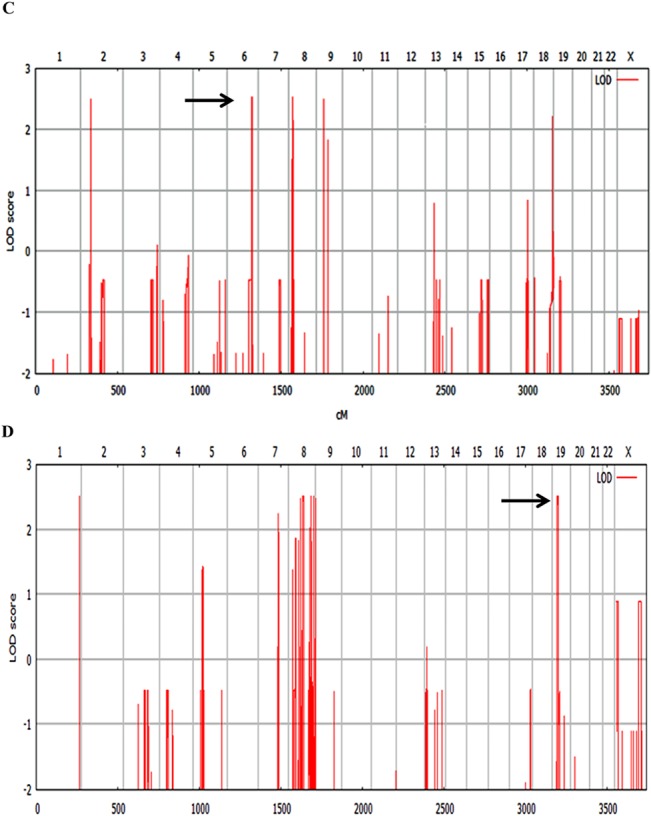
Linkage analysis results. (A) Parametric linkage analysis for family 9000105: indicates peaks (Lod≥2.5) including an IBD (Identical by descend) segment on chromosome 6. (B) Parametric linkage analysis for family 9000114: indicates peaks (Lod≥2.5), including a 5.6 Mb interval on chromosome 19.

**Fig 4:**
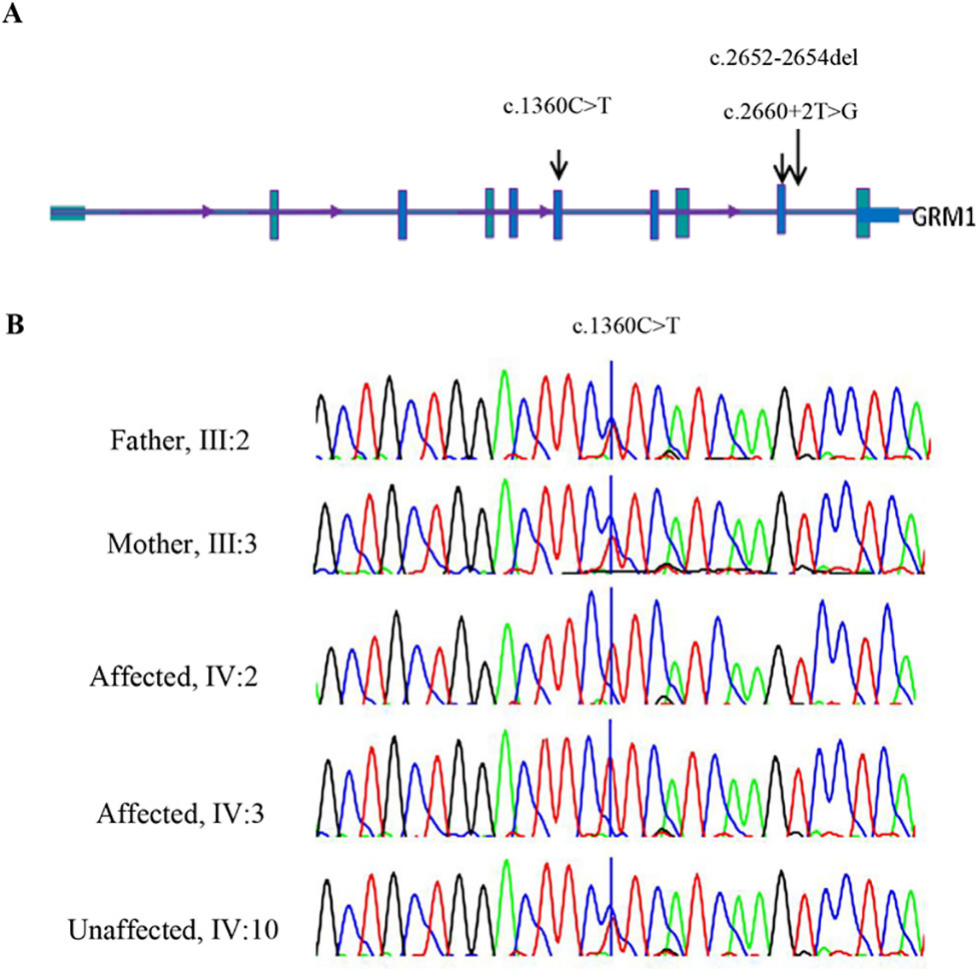
The candidate mutation in the *GRM1* gene and its segregation within the family. (A) Scheme of the *GRM1* gene structure; arrows indicated the mutation identified in this study (c.1360C>T) and those published by Guerguelcheva et al. (B). Sequence chromatograms showing the complete segregation of the missense mutation c.1360C>T in family 9000105 in the patients.

Genotype analysis of the family 9000105 revealed five intervals of autozygosity with a LOD score ≥2.5 (Fig 3A and S1 Table). Whole exome sequencing (WES) was then performed using one of the affected individuals (IV.1). After filtering the variants among candidate variants, a homozygous missense variant in *GRM1*gene [chr6. hg19: g.146673559C>T; NM_000838.2: c.1360C>T; p.Leu454Phe] (coordinates used in hg19) was ranked as potentially causative variant in this family. The candidate mutation in the *GRM1* gene was located on a 5 Mbp identical by descend (IBD) segment with a LOD score of 2.7 on chromosome 6q24.2–24.3, flanked by heterozygous SNPs rs4473877: T>C and rs2341768:C>T (Figs 3 and 4).

**Fig 5:**
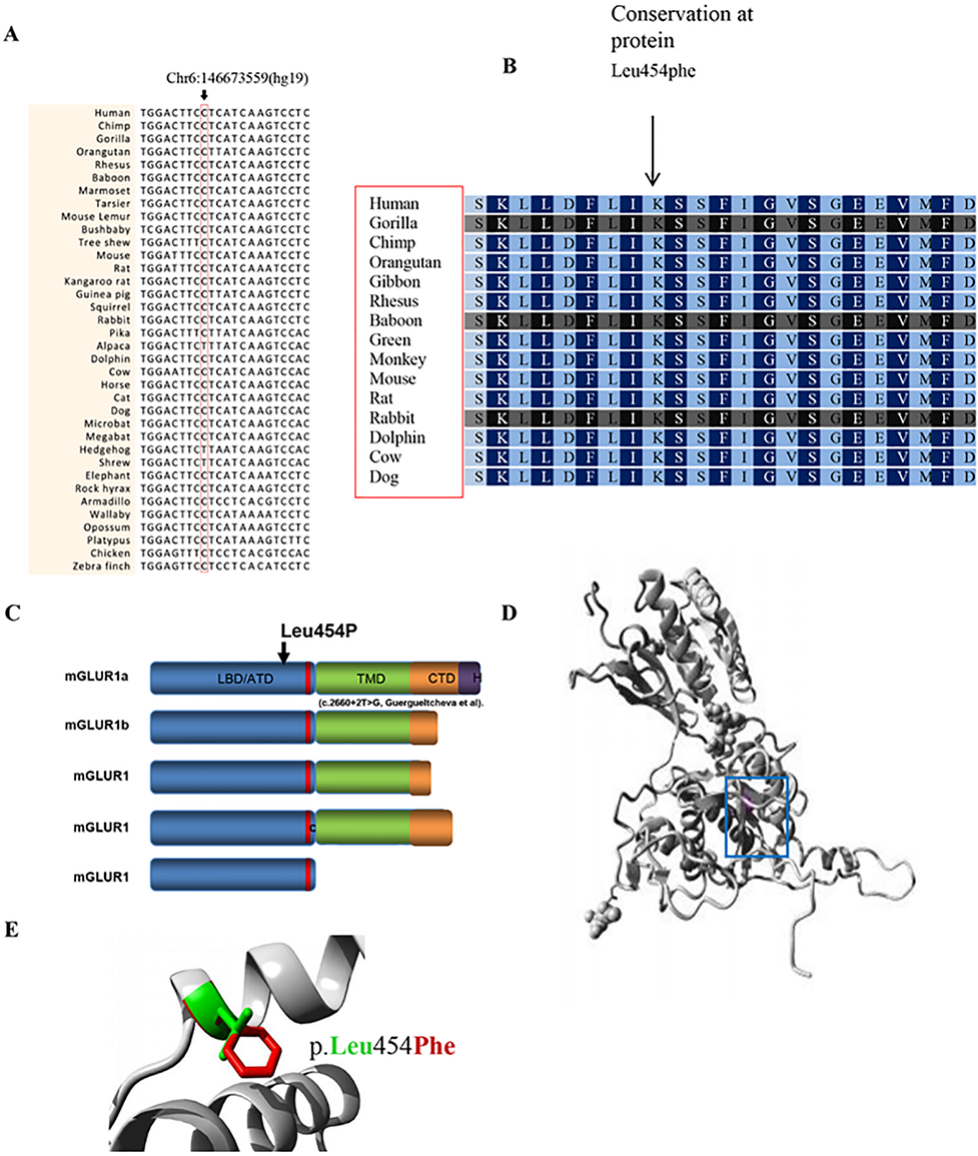
mGluR1 conserved protein isoforms with *In silico* analysis of *GRM1* missense substitution. (A) Partial nucleotide sequence alignment of human *GRM1* and its homologues in the animal kingdom (CLUSTAL2.1). The position of the missense mutation in family 9000105 is indicated by an arrow. (B) Partial amino acid sequence alignment of the human mGluR1 protein and its homologues in the animal kingdom. (C) mGluR1 protein isoforms. Domain LBD/ATD = ligand binding domain/amino terminal domain, C = cysteinerich domain in red domain, TMD = transmembrane domain, CTD = C-terminal domain, H = Homer 1-binding motif.^**42**^ (D) Ribbon presentation of the mGluR1 protein. The protein is colored grey, the side chain of the mutated residue, p.Leu454Phe, is colored magenta and shown as small balls. (E) Close-up of the p.Leu454Phe mutation. The protein is colored grey, the side chains of both the wild-type and the mutant residue are shown and colored green and red, respectively. The p.Leu454Phe position is indicated.

The p.Leu454Phe variant is located in the ligand binding domain (LBD), which is present in all mGluR1 protein isoforms. The amino acid Leu454 has a high PhyloP score and is conserved across evolution through the animal kingdom, suggesting an important role in the normal functioning of the protein. *In silico* analysis of GRM1 missense substitution using PolyPhen and SIFT predict this change as being "probably damaging" and "damaging". We used project HOPE [25] to perform 3D modelling of p.Leu454Phe change. The wild type residue is located in an α-helix. The mutation converts the wild type residue (leucine) into phenylalanine that does not prefer α-helices in its secondary structure. This residue is part of the so-called extracellular LBD and is buried in the core of the domain itself. The phenylalanine residue is larger in size than leucine residue and probably could not fit in the domain therefore resulting in protein folding problems (Fig 5).

**Fig 6:**
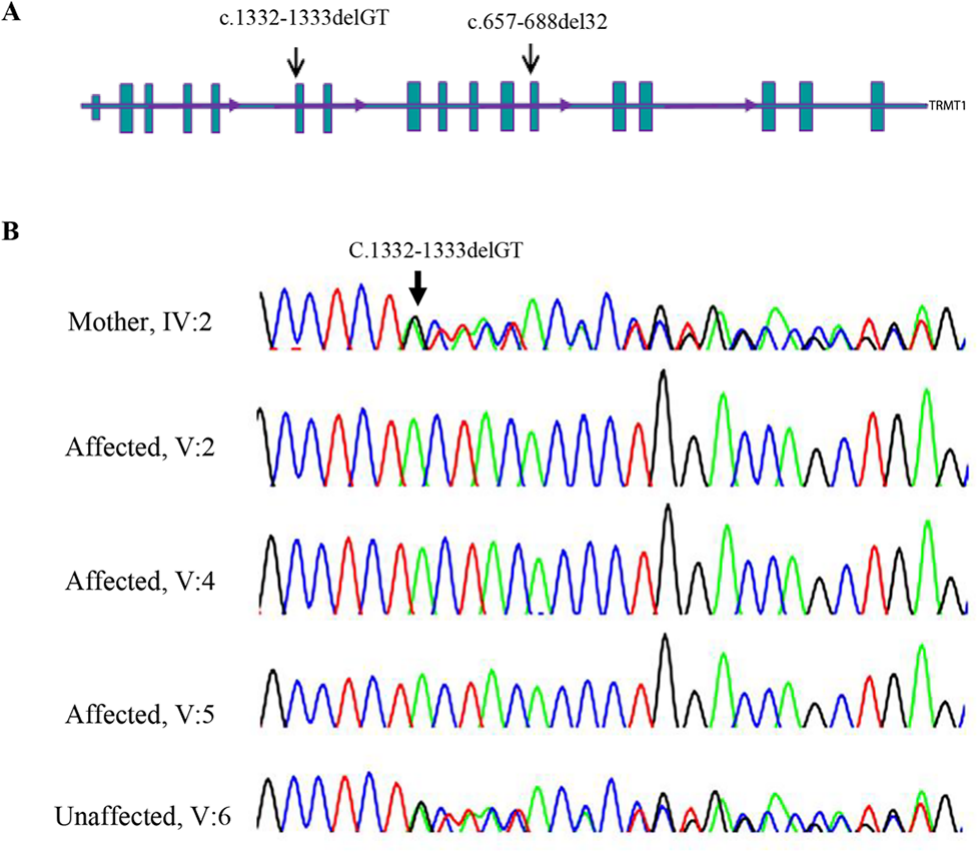
The novel *TRMT1* gene alteration and its complete segregation in 9000114 family. (A) Scheme of the *TRMT1* gene structure; arrows indicated the mutation identified in this study (c.1332_1333delGT) and the one published by Najmabadi et al. (see text). (B) Sequence chromatograms showing the complete segregation of the homozygous c.1332-1333delGT in 900114 family in the patients (5.2, 5.4 and 5.5), the healthy sibling (5.6) and mother (4.2) are heterozygous for deletion.

Genotyping and linkage analysis of family 9000114 uncovered four homozygous genomic intervals with a LOD score ≥2.5 (Fig 3B and S1 Table). Following the same approach used for the family described above, WES was performed using DNA from individual V.2. After filtering and prioritization from candidate mutations, we ranked a homozygous 2 bp deletion in *TRMT1* gene [chr19.hg19: g.13220259_13220260delAC; NM_001136035.2: c.1332_1333delGT; p.Tyr445Leufs*28] (coordinates used in hg19) as responsible variant which impact the function of gene. The *TRMT1* alteration lies in a 5.6 Mbp homozygous linkage interval on chromosome 19p13.12–13.2 (LOD score of 2.5) flanked by the heterozygous SNPs rs7254567: A>G and rs17750057: C>A. (Figs 3 and 6).

Sanger sequencing analysis confirmed co-segregation of the two recent variants with the disease in the family and also the absence of the each variant in 100 individuals of ethnic specific Iranian controls (Figs 4 and 6).

## Conclusion

In theory, it is thought that many causative ID genes could participate in the common biological processes and pathways that elucidate the genetic basis of cognitive functions [6]. Other important genes could also have an impact on the chromatin modification process [26]. However, several studies have emphasized the role of synaptic- or neuron-specific gene defects in the aetiology of ID [27, 28].

Our understanding of the metabotropic glutamate receptors role in the central nervous system has grown rapidly in the past few decades [29]. Glutamatergic neurotransmission is involved in most aspects of normal brain function and can be perturbed in many neuropathological conditions [30,31,32,33,34,35,36,37]. GRM1 is a G protein coupled receptor for glutamate (GPCR). Interesting electrophysiological experiments in mGluR1 –/–mice show abnormal long-term potentiation and point to a critical role for mGluR1 in synaptic plasticity, memory, and learning in other aspects [38]. Conversely, specific removal of mGluRs in the hippocampus causes deficits in long-term depression in the cerebellum, and these mice have gait disabilities [39, 40].

Among our comprehensive cohort of Iranian autosomal recessive ID (ARID) families, this was the first time we identified a mutation in the *GRM1* gene. The similarities between the symptoms of individuals with congenital ataxia and additional features and those presented by patients with a SCAR13 phenotype (Guergultcheva et al., 2012), delayed infancy development, mild-to-profound ID with poor or absent speech, and gait and stance ataxia have been distinguished (Table 1). In silico analysis and 3D modelling of the mutant protein suggest a probable pathogenic role of the p.Leu454Phe mutation, which is located in a region encoding the LBD of the mGluR1 protein. All spontaneous mutations occurred in the LBD of the mGluR1, suggesting a critical role for this domain in the ataxic gait problems [41].

The binding of extracellular ligands, that is, peptides and neurotransmitters, to the LBD of mGluR1 induces a conformational change in the G protein coupled receptors (GPCRs), which can precipitate in a variety of signalling responses, including activation of G proteins, which are composed of three subunits α, β and γ, and consequent downstream signalling cascades. Hence, activation of G proteins has an effect on various functional agents such as transcription factors, ion channels and enzymes. Inactivation of G proteins resulted in the exchange of GTP for GDP [29]. Extensive studies to discover the mechanism of the activation and function of GPCRs as well as the interacting protein models that modulate them, specifically the many novel ligands, have also contributed to finding therapeutic perspectives in neurologic and psychiatric disorders.

In one of the families reported in this study, a frameshift alteration in the *TRMT1* gene resulted in an allelic variant that lies within the TRM functional domain, which is responsible for the enzymatic activity of the protein. This frameshift variant led to a premature termination codon (PTC) that affected the splicing of all *TRMT1* splice variants, which probably undergo degradation mediated by nonsense-mediated mRNA decay (NMD).

Basel-Vanagaite et al. in 2003 identified a new locus in the chromosomal region 19p13.12-p13.2, which contains a critical 2.4 MB region between the proximal D19S547 and distal D19S1165 loci, which resulted in autosomal recessive non-syndromic mental retardation in four consanguineous families of Israeli Arab origin [42]. Surprisingly, the *TRMT1* gene maps onto the aforementioned region.

Interestingly, mutations in two genes encoding RNA-methyltransferases, *NSUN2* and *FTSJ1*, are functionally similar to *TRMT1* and are associated with perturbing the post-translation of tRNAs, causing the ID phenotype. Co-immunoprecipitation assays have also shown protein-protein interactions between NSUN2 and FTSJ1 (IntAct interaction database), suggesting a common functional pathway [26, 14]. However, a functional study with *TRMT1* has not yet been performed to assess its role in human development; it would be beneficial to research in the future.

As mentioned above, this is the second ARID family in which *TRMT1* defect has been identified. The first family reported by Najmabadi et al.2011, consisted of five offspring, two of whom were affected by moderate-to-severe ID. Upon examination, they showed pes planus and slight facial dysmorphism, including protruding ears, narrowing palpebral fissure and broad eyebrows. Spasticity in the lower limbs started at puberty and progressed gradually. The clinical findings in our study are consistent with the phenotype of the family described by Najmabadi et al., particularly the involvement of the upper and lower limbs and spasticity progression. The clinical features are summarized in Table 2. However, we did not observe any obvious abnormal findings in the brain MRIs, though the previous study revealed dilated 4^th^ ventricle and cerebellar atrophy symptoms.

In this case and other similar cases, we believe that the identification of multiple patients with overlapping clinical features and allelic mutations is suggestive of a pathogenic role of the identified variants in the same gene. Our previous study [10] highlights the potential of linkage analysis as a valid tool to map disease genes, in combination with deep sequencing strategy, contributes to mutation discovery. Our findings also reveal that Iranian heterogeneous population in combination with consanguineous marriages would lead to identification of disease-associated genes.

## Supporting Information

S1 Table Data of the homozygote intervals. Information of the homozygote intervals within the 9000105 and 9000114 families based on the autozygosity mapping.
(DOCX)

S2 Table List of variant identified in M9000105. (XLS)

S3 Table List of variant identified in M9000114. (XLSX)

## References

[1] Roeleveld N, Zielhuis GA, Gabreels F (1997) The prevalence of mental retardation: a critical review of recent literature. Dev Med Child Neurol, 39,125–32. 9062428 10.1111/j.1469-8749.1997.tb07395.x

[2] Leonard H, Wen X (2002) The epidemiology of mental retardation: challenges and opportunities in the new millennium. Ment Retard Dev Disabil Res Rev, 8:117–34. 12216056 10.1002/mrdd.10031

[3] McLaren J, Bryson SE) 1987) Review of recent epidemiological studies of mental retardation: prevalence, associated disorders, and etiology. Am J Ment Retard, 92:243–54. 3322329

[4] Musante H, Ropers HH (2014) Genetics of recessive cognitive disorders. Trends Genet, 30910:32–9. 10.1016/j.tig.2013.09.008 24176302

[5] Hoodfar E, Teebi AS (1996) Genetic referrals of Middle Eastern origin in a western city: inbreeding and disease profile. J Med Genet, 33, 212–5. 8728693 10.1136/jmg.33.3.212 PMC1051869

[6] Van Bokhoven H (2001) Genetic and epigenetic networks in intellectual disabilities. Ann Rev Genet, 45:81–104. 10.1146/annurev-genet-110410-132512 21910631

[7] Rauch A, Hoyer J, Guth S, Zweier C, Kraus C, Becker C, et al (2006) Diagnostic yield of various genetic approaches in Patients with unexplained developmental delay or mental retardation. Am J Med Genet A, 140:2063–74. 16917849 10.1002/ajmg.a.31416

[8] Najmabadi H, Motazacker MM, Garshasbi M, Kahrizi K, Tzschach A, Chen W, et al (2007) Homozygosity mapping in consanguineous families reveals extreme heterogeneity of non-syndromic autosomal recessive \ mental retardation and identifies 8 novel gene loci. Hum Genet, 121:43–8. 17120046 10.1007/s00439-006-0292-0

[9] Kuss AW, Garshasbi M, Kahrizi K, Tzschach A, Behjati F, Darvish H, et al (2011) Autosomal recessive mental retardation: homozygosity mapping identifies 27 single linkage intervals, at least 14 novel loci and several mutation hotspots. Hum Genet,129:141–8. 10.1007/s00439-010-0907-3 21063731

[10] Najmabadi H, Hu H, Garshasbi M, Zemojtel T, Abedini SS, Chen W, et al (2011) Deep sequencing reveals 50 novel genes for recessive cognitive disorders. Nature, 478:57–63. 10.1038/nature10423 21937992

[11] Desai A, Burnett JP, Mayne NG (1995) Cloning and expression of a human metabotropic glutamate receptor 1 alpha: enhanced coupling on co- transfection with a glutamate transporter. Mol Pharmacol, 8:648–57. 7476890

[12] Liu J, Straby KB (2000) The human tRNA (m22G26) dimethyltransferase: functional expression and characterization of a cloned hTRM1 gene. Nucleic Acids Res. 28:3445–51. 10982862 10.1093/nar/28.18.3445 PMC110725

[13] Freude K, Hoffmann K, Jensen LR, Delatycki MB, des Portes V, Moser B, et al (2004) Mutations in the FTSJ1 gene coding for a novel S-adenosylmethionine-binding protein cause nonsyndromic X-linked. Am J Hum Genet. 75:305–9. 15162322 10.1086/422507 PMC1216064

[14] Khan MA, Rafiq MA, Noor A, Hussain S, Flores JV, Rupp V, et al (2012) Mutation in NSUN2, which encodes an RNA methyltransferase, causes autosomal-recessive intellectual disability. Am J Hum Genet, 90, 856–63. 10.1016/j.ajhg.2012.03.023 22541562 PMC3376419

[15] Li C, Wong WH (2001) Model-based analysis of oligonucleotide arrays: Expression index computation and outlier detection. Proc Natl Acad Sci USA, 98:31–6. 11134512 10.1073/pnas.011404098 PMC14539

[16] Seelow D, Schuelke M, Hildebrandt F, Nürnberg P (2009) Homozygosity Mapper an interactive approach to homozygosity mapping. Nucleic Acids Res, 37:W593–9. 10.1093/nar/gkp369 19465395 PMC2703915

[17] Ruschendorf F, Nurnberg P (2005) ALOHOMORA: A tool for linkage analysis using 10K SNP array data. Bioinformatics, 21:2123–5. 15647291 10.1093/bioinformatics/bti264

[18] O’Connell JR, Weeks DE (1998) PedCheck: A program for identification of genotype incompatibilities in linkage analysis. Am J Hum Genet, 63:259–66. 9634505 10.1086/301904 PMC1377228

[19] Abecasis GR, Cherny SS, Cookson WO, Cardon LR (2001) GRR: Graphical representation of relationship errors. Bioinformatics, 17:742–3. 11524377 10.1093/bioinformatics/17.8.742

[20] Abecasis GR, Cherny SS, Cookson WO, Cardon LR (2002) Merlin–rapid analysis of dense genetic maps using sparse gene flow trees. Nat Genet 30:97–101. 11731797 10.1038/ng786

[21] Durbin RM (2010) A map of human genome variation from population-scale sequencing. Nature, 467:1061–73. 10.1038/nature09534 20981092 PMC3042601

[22] Li Y, Vinckenbosch N, Tian G, Huerta-Sanchez E, Jiang T, Jiang H, et al (2010) Resequencing of 200 human exomes identifies an excess of lowfrequency non-synonymous coding variants. Nat Genet, 42:969–72. 10.1038/ng.680 20890277

[23] Berg JS, Khoury MJ, Evans JP (2011) Deploying whole genome sequencing in clinical practice and public health: meeting the challenge one bin at a time. Genet Med, 13:499–504. 10.1097/GIM.0b013e318220aaba 21558861

[24] Neale BM, Kou Y, Liu L, Ma/'ayan A, Samocha KE, Sabo A, et al (2012) Patterns and rates of exonic de novo mutations in autism spectrum disorders. Nature, 485:242–5. 10.1038/nature11011 22495311 PMC3613847

[25] Venselaar H, Beek T, Kuipers R, Hekkelman ML, Vriend G (2010) Protein structure analysis of mutations causing inheritable diseases. An e-Science approach with life scientist friendly interfaces. BMC bioinformatics, 11:548 10.1186/1471-2105-11-548 21059217 PMC2992548

[26] Kleefstra T, Kramer JM, Neveling K, Willemsen MH, Koemans TS, Vissers LE, et al (2012) Disruption of an EHMT1-associated chromatin-modification module causes intellectual disability. Am J Hum Genet, 91:73–82. 10.1016/j.ajhg.2012.05.003 22726846 PMC3397275

[27] Hamdan FF, Gauthier J, Araki Y, Lin DT, Yoshizawa Y, Higashi K, et al (2011) Excess of de novo deleterious mutations in genes is associated with glutamatergic systems in nonsyndromic intellectual disability. Am J Hum Genet, 88:306–16. 10.1016/j.ajhg.2011.02.001 21376300 PMC3059427

[28] Laumonnier F, Cuthbert PC, Grant SG (2007) The role of neuronal complexes in human X-linked brain diseases. Am J Hum Genet, 80:205–20. 17236127 10.1086/511441 PMC1785339

[29] Niswender CM, Conn PJ (2010) Metabotropic glutamate receptors: Physiology, pharmacology and disease. Annu Rev Pharmacol Toxicol, 50:295–322. 10.1146/annurev.pharmtox.011008.145533 20055706 PMC2904507

[30] Coutinho V, Knopfel T (2002) Metabotropic glutamate receptors: electrical and chemical signaling properties. Neuroscientist, 8:551–61. 12467377 10.1177/1073858402238514

[31] Mannaioni G, Marino MJ, Valent O, Traynelis SF, Conn PJ (2001) Metabotropic glutamate receptors 1 and 5 differentially regulate CA1 pyramidal cell function. Neurosci, 21:5925–34. 10.1523/JNEUROSCI.21-16-05925.2001 PMC6763150 11487615

[32] Conn PJ, Pin JP (1997) Pharmacology and functions of metabotropic glutamate receptors. Annu Rev Pharmacol Toxicol, 37:205–37. 9131252 10.1146/annurev.pharmtox.37.1.205

[33] Valenti O, Conn PJ, Marino MJ) 2002) Distinct physiological roles of the G-coupled metabotropic glutamate receptors co-expressed in the same neuronal populations. J Cell Physiol, 191:125–37. 12064455 10.1002/jcp.10081

[34] Pinheiro PS, Mulle C) 2008) Presynaptic glutamate receptors: physiological functions and mechanisms of action. Nat Rev Neurosci, 9:423–36. 10.1038/nrn2379 18464791

[35] Bellone C, Luscher C, Mameli M (2008) Mechanisms of synaptic depression triggered by metabotropic glutamate receptors. Cell Mol Life Sci, 65:2913–23. 10.1007/s00018-008-8263-3 18712277 PMC11131865

[36] Anwyl R) 1999) Metabotropic glutamate receptors: electrophysiological properties and role in plasticity. Brain Res Rev, 29:83–120. 9974152 10.1016/s0165-0173(98)00050-2

[37] Kullmann DM, Lamsa K (2008) Roles of distinct glutamate receptors in induction of anti- Hebbia longterm potentiation. J Physiol, 586:1481–86. 10.1113/jphysiol.2007.148064 18187472 PMC2375711

[38] Gil-Sanz C, Delgado-Garcıa JM, Fairén A, Gruart A (2008) Involvement of the mGluR1receptor in hippocampal synaptic plasticity and associative learning in behaving mice. Cereb Cortex, 18:165363. 10.1093/cercor/bhm193 18024992

[39] Alba A, Kano M, Chen C, Stanton ME, Fox GD, Herrup K, et al (1994) Deficient cerebellar long-term depression and impaired motor learning in mGluR1 mutant mice. Cell, 79:377–88. 7954803

[40] Aiba A, Chen C, Herrup K, Rosenmund C, Stevens CF, Tonegawa S (1994) Reduced hippocampal longterm potentiation and context-specific deficit in associative learning in mGluR1 mutant mice. Cell, 79:365–75. 7954802 10.1016/0092-8674(94)90204-6

[41] Sachs AJ, Schwendinger JK, Yang AW, Haider NB, Nystuen AM (2007) The mouse mutants recoil wobbler and nmf373 represent a series of Grm1 mutations. Mamm Genome.,18:749–56. 17934773 10.1007/s00335-007-9064-y

[42] Basel-Vanagaite L, Alkelai A, Straussberg R, Magal N, Inbar D, Mahajna M, et al (2003) Mapping of a new locus for autosomal recessive non-syndromic mental retardation in the chromosomal region 19p13.12-p13.2: further genetic heterogeneity. J Med Genet, 40:729–32. 14569116 10.1136/jmg.40.10.729 PMC1735276

