# Supplementary material for: The Role of a Novel TRMT1 Gene Mutation and Rare GRM1 Gene Defect in Intellectual Disability in Two Azeri Families
Source: PLoS One. 2015 Aug 26;10(8):e0129631. doi: 10.1371/journal.pone.0129631 (PMC4550366; doi:10.1371/journal.pone.0129631)
Supplement: S1 Table — Information of the homozygote intervals within the 9000105 and 9000114 families based on the autozygosity mapping. (DOCX) [file pone.0129631.s001.docx]

**S1 Table .Data of the homozygote intervals:**

Information of the homozygote intervals within the 9000105 and 9000114 families based on the autozygosity mapping.

|  | Flanking heterozygous SNP markers | Chromosomal band | Size | Lod -score |
| --- | --- | --- | --- | --- |
| 9000105 family | rs6740241-6722589 | Chr.2p22.2 | 95Kb | 2.5 |
|  | 4473877-2341768 | Chr.6q24.2-25.1 | 5Mb | 2.529 |
|  | 10087914-11774569 | Chr.8p23.1 | 53.7Kb | 2.529 |
|  | 1888992-16812209 | Chr:9p21.3 | 57kb | 2.489 |
|  | 12103990-12964419 | Chr.18q23 | 281Kb | 2.204 |
| 9000114 family | 12123018-484459 | Chr.1q44 | 35.5Kb | 2.529 |
|  | 38760-10242441 | Chr.7q31.1 | 350Kb | 2.46 |
|  | 5891696-2693453 | Chr.8q12.1 | 45Kb | 2.48 |
|  | 4738111-7002599 | Chr.8q13.3 | 164Kb | 2.529 |
|  | 002599-4321959 | Chr.8q21.11 | 8.4Mb | 2.529 |
|  | 844673-12680265 | Chr.8q24.21 | 146Kb | 2 |
|  | 2549659-11787498 | Chr.8q24.23 | 195Kb | 2.529 |
|  | 730453-35253016 | Chr.8q24.23 | 488Kb | 2.529 |
|  | 11248216-6599558 | Chr.8q24.3 | 126Kb | 2.509 |
|  | 7254567-17750057 | Chr.19p13.12-13.2 | 5.6Mb | 2.529 |
